# Imposed Optical Defocus Induces Isoform-Specific Up-Regulation of TGFβ Gene Expression in Chick Retinal Pigment Epithelium and Choroid but Not Neural Retina

**DOI:** 10.1371/journal.pone.0155356

**Published:** 2016-05-23

**Authors:** Yan Zhang, Suravi Raychaudhuri, Christine F. Wildsoet

**Affiliations:** Center for Eye Disease & Development, Vision Science Program & School of Optometry, University of California, Berkeley, California, United States of America; Wenzhou Medical University, CHINA

## Abstract

**Purpose:**

This study investigated the gene expression of TGFβ isoforms and their receptors in chick retina, retinal pigment epithelium (RPE), and choroid and the effects of short-term imposed optical defocus.

**Methods:**

The expression of TGFβ isoforms (TGF-β1, 2, 3) and TGFβ receptors (TGFBR1, 2, 3) was examined in the retina, RPE, and choroid of young White-Leghorn untreated chicks (19 days-old). The effects on the expression of the same genes of monocular +10 and -10 D defocusing lenses, worn for either 2 or 48 h by age-matched chicks, were also examined by comparing expression in treated and untreated fellow eyes. RNA was purified, characterized and then reverse transcribed to cDNA. Differential gene expression was quantified using real-time PCR.

**Results:**

All 3 isoforms of TGFβ and all 3 receptor subtypes were found to be expressed in all 3 ocular tissues, with apparent tissue-dependent differences in expression profiles. Data are reported as mean normalized expression relative to GAPDH. Sign-dependent optical defocus effects were also observed. Optical defocus did not affect retinal gene expression but in the RPE, TGF-β2 expression was significantly up-regulated with +10 D lenses, worn for either 2 h (349% increase ± 88%, *p* < 0.01) or 48 h (752% increase ± 166%, *p* < 0.001), and in the choroid, the expression of TGF-β3 was up-regulated with -10 D lenses, worn for 48 h (147% increase ± 9%, *p* < 0.01).

**Conclusions:**

The effects of short term exposure to optical defocus on TGFβ gene expression in the RPE and choroid, which were sign-dependent and isoform specific, provide further supporting evidence for important roles of members of the TGFβ family and these two tissues in local signal cascades regulating ocular growth.

## Introduction

Refractive errors are a leading contributor to the global burden of eye disease, dominating the eye disease profiles of high-, middle-, and low-income countries.[1] Refractive errors occur when there is a mismatch between the focusing power of the eye and its axial length. This mismatch may result in the image plane for distant objects lying anterior to the retina, as in myopia, or posterior to the retina, as in hyperopia. Increased interest in how refractive errors develop stems from the recent dramatic rise in the prevalence of myopia world-wide, now at epidemic levels in many East Asian countries.[2–5] In addition to the economic costs associated with correcting myopia and decreased quality of life experienced by individuals with uncorrected myopia, associated pathological complications, including glaucoma, retinal detachment and chorioretinal atrophy, carry risks of permanent loss of vision.[6]

A combination of genetic and environmental factors is thought to contribute to the development of human myopia.[7] While infants frequently exhibit refractive errors, they are typically corrected during early development through adjustments to eye length by a process known as emmetropization.[8] Studies involving animal models provide conclusive evidence for an active, defocus-driven emmetropization mechanism, and thus refractive errors that persist or emerge during development may be considered a result of failure of this regulatory mechanism.[9, 10]

Of the animal models used to study eye growth regulation, the chick model has provided valuable insights into underlying mechanisms. Both form deprivation and imposed optical defocus, applied during early development, reliably induce refractive errors in this model.[11, 12] Importantly, the effect of the defocus is sign-dependent; imposed hyperopic defocus, achieved with negative lenses, accelerates eye growth while imposed myopic defocus, achieved with positive lenses, inhibits it. Refractive errors—myopia and hyperopia—are the byproducts of the resulting mismatches between the refracting power of the eye and its altered length. Lesioning studies aimed at identifying elements of the underlying signal pathway point to a local ocular circuit, since neither cutting the optic nerve, to sever the link between the retina and brain, nor cutting the ciliary nerve, to eliminate active accommodation, prevent the experimental induction of refractive errors.[13–16]

The local signal pathway underlying ocular growth regulation was the target of the study reported here. The presumed elements are the retina, onto which images of the external world project, the choroid and sclera, which represent the outer structural layers of the vitreous chamber, and the intervening retinal pigment epithelium (RPE). Experimentally-induced changes involve both the choroid, which has been shown to thicken with imposed myopic defocus and thin in response to hyperopic defocus, and the overlying sclera, which undergoes altered growth and/or remodeling, depending on the species.[17, 18] It is assumed that signal transduction begins in the retina and as the RPE is sandwiched between the retina and the choroid, it likely plays a key role in relaying signals between the retina and choroid/sclera.[19] Although only one cell layer thick, the RPE is rich in receptors for signaling molecules that have already been implicated in ocular growth regulation, including dopamine, acetylcholine, vasoactive intestinal peptide (VIP), and glucagon.[19–23] Furthermore, there are reports of differential gene expression in the RPE linked to experimentally-induced myopia and hyperopia.[19, 24–27]

The transforming growth factor β (TGFβ) superfamily represents a large family of structurally related multifunctional growth factors,[28, 29] which includes various isoforms of TGFβ as well as of bone morphogenetic protein (BMP), some of which have already been linked to ocular growth regulation.[24, 25] TGFβ isoforms (TGF-β1, TGF-β2, and TGF-β3) are dimeric cytokines secreted by many cell types and integral to a wide spectrum of physiological and pathological processes, including embryonic development, organogenesis, extracellular matrix remodeling, wound healing, immune modulation and cancer progression.[28, 29] The biological effects of TGFβs are mediated through type I and type II receptors (TGFBR1 and TGFBR2), with a third receptor, TGFBR3, functioning as an accessory for ligand presentation to TGFBR2.

TGFβs have already been targeted in some myopia studies, which were largely focused on scleral changes. In the tree shrew sclera, gene expression of all three isoforms of TGFβ were found to be down-regulated after 1 and 5 days of form-deprivation, which induces myopia,[30, 31] and likewise, the expressions of TGF-β1 and TGF-β2 were reported to be down-regulated after 4 days of -5 D lens treatment.[32] The opposite result, up-regulation of TGF-β2 gene expression, has been reported in the cartilaginous layer of the chick sclera after 24 h of +7 D lens treatment.[33] In the tree shrew sclera, TGFBR3 expression was also found to be up-regulated with the above 4-day, -5 D lens treatment.[32] Expression changes in the RPE were not investigated in these studies.

Providing the rationale for the study described here, TGFβs have been shown to be expressed in the RPE, as well as retina and choroid of human eyes.[34–37] In a more closely related study, we have reported differential regulation of TGF-β2 transcripts in the RPE of chicks treated long-term (38 days) with -15 D lenses,[38] although in a recent study in tree shrew, no change in gene expression was observed for TGF-β1, 2 and 3, in either retina or choroid, in response to form deprivation.[39] However, to-date, there has been no comprehensive gene expression study of the TGFβ isoforms and receptors in chick ocular tissues critical to ocular growth regulation—retina, RPE, and choroid, either in normal eyes or eyes subjected to short-term optical defocus, when eyes can be expected to be actively responding to their altered visual experience. The study described here was intended to cover this knowledge gap for the chick model. We established gene expression profiles for all three TGFβ isoforms (TGF-β1, TGF-β2, and TGF-β3) and their three receptors (TGFBR1, TGFBR2, and TGFBR3) in chick retina, RPE, and choroid, and examined the effects on gene expression of short-term exposure to imposed optical defocus.

## Materials and Methods

### Animals & Lens Treatments

White-Leghorn chicks were hatched from fertilized eggs obtained from University of California, Davis (Davis, CA), and raised under a 12 h light/12 h dark cycle, with food and water ad libitum, in an Animal Facility at University of California, Berkeley (Berkeley, CA). Experiments were conducted according to the ARVO Statement for the Use of Animals in Ophthalmic and Vision Research and approved by the Animal Care and Use Committee (ACUC) at University of California, Berkeley (Berkeley, CA). Gene expression studies made use of both untreated 19 days-old chicks and age-matched chicks that wore monocular lenses, either +10 or -10 D, for 2 or 48 h. Untreated contralateral (fellow) eyes served as controls in the latter experiment. Data for the four treatment groups represent the compilation of two or three independent repetitions of this experiment (3–4 chicks in each experiment). Untreated chicks served as additional controls.

### Tissue Sample Collection for Gene Expression Studies

Retina, RPE, and choroid samples were collected from both eyes of lens-wearing and untreated chicks. The method of sample collection was as described previously.[24, 25] Briefly, chicks were sacrificed with a guillotine, eyes enucleated and then retina, RPE, and choroid isolated. All three ocular tissues were lysed with RLT buffer from RNeasy Mini kits (Qiagen, Valencia, CA). Samples were immediately frozen and stored at -80°C for later use.

### RNA Purification and Reverse Transcription

For retina and RPE samples, total RNA was purified using RNeasy Mini kits, while RNA from choroid samples was purified using an RNeasy Fibrous Tissue Mini Kit (Qiagen). For all RNA samples, on-column DNase digestion was then performed and RNA concentration and optical density ratio of A260/A280 measured with a spectrophotometer (NanoDrop 2000, NanoDrop Technologies, Inc., Wilmington, DE). RNA samples were then reverse transcribed to cDNA (SuperScript III First-Strand Synthesis System for RT-PCR, Invitrogen, Carlsbad, CA).

### Real-Time PCR

Primers were designed using Primer Express 3.0 (Applied Biosystems, Foster City, CA. Table 1). The efficiency (E) of each primer was calculated using Eq (1), with the slope of the standard curves generated using ten-fold serial dilutions of cDNA. The amount of cDNA used in each PCR reaction varied according to gene expression levels in the different tissues. QuantiTect SYBR Green PCR Kits (Qiagen) were used for mRNA amplification with a StepOnePlus Real-Time PCR System (Applied Biosystems).

**Table 1:** Details of primers used in assays for the isoforms of chick TGF-βs and TGF-β receptors.

Gene	NCBI Access Number	Sequences (5’-3’)	Efficiency	Amplicon
TGF-β1	JQ423909.1	Forward: 5’-ACATCGACTTCCGCAAGGAT-3’	100%	69 bp
		Reverse: 5’-AGAAGTTGGCCATATAACCTTTGG- 3’		
TGF-β2	NM_001031045.2	Forward: 5’-GCTGCGTGTCCCAGGATTT-3’	95.1%	60 bp
		Reverse: 5’-TGGGTGTTTTGCCAATGTAGTAGA-3’		
TGF-β3	NM_205454.1	Forward: 5’-TGATGATGCTACCCCCACATC-3’	99.4%	141 bp
		Reverse: 5’-CCTGTCGGAAGTCAATGTAAAGAG-3’		
TGFBR1	NM_204246.1	Forward: 5’- CAATTCCAACACCAGGTCCTACT-3’	100%	66 bp
		Reverse: 5’- CTGCCAGTTCCACAGGTCCTA-3’		
TGFBR2	NM_205428.1	Forward: 5’-CAGATGTGTACTCCATGGCTTTG-3’	100%	82 bp
		Reverse: 5’-GGCTCATACTCTTTCACTTCTCCAA-3’		
TGFBR3	NM_204339.1	Forward: 5’-CTGCAGGCAAGCGGCTATAC-3’	100%	68 bp
		Reverse: 5’-TTCATCCTGGCTTTGCAAGA-3’		

Mean normalized expression (MNE) values were calculated for target genes using Eq (2), using as the reference gene, glyceraldehyde 3-phosphate dehydrogenase (GAPDH), with primer efficiency as reported previously.[25] The stability of GAPDH expression across different treatment conditions was assessed by comparing the expression of GAPDH/total RNA in retinal and choroidal samples from untreated, treated, and fellow eyes, replicating a previous study design undertaken to establish the stability of GAPDH expression in RPE.[24] For each of the 4 treatment conditions, mean mRNA expression levels were then calculated using Eq (3),[25] with induced changes expressed as percent change, calculated using Eq (4). Data for right and left eyes of untreated chicks were similarly compared. Melt curves were examined to verify the yield of single peak products and all real-time PCR reactions were performed in triplicate.

$$E=10^{\left(-1/slope\right)}$$ Eq 1

$$MNE=\frac{{\left(E_{reference}\right)}^{{CT}_{reference,mean}}}{{\left(E_{target}\right)}^{{CT}_{target,mean}}}$$ Eq 2

$$mRNA\;levels=\frac{1}{N}\;\sum_{i=1}^{N}{MNE}_{i}$$ Eq 3

$$Percent\;Change=\frac{1}{N}\;\sum_{i=1}^{N}\frac{{Treatment}_{i}}{{Fellow}_{i}}\times 100\%\;\text{for treated birds or}\;Percent\;Change=\frac{1}{N}\;\sum_{i=1}^{N}\frac{{Right}_{i}}{{Left}_{i}}\times 100\%\;\text{for untreated birds}$$ Eq 4

### Statistical Analyses

Paired Student’s *t*-tests were used to compare lens-treated eyes with their fellows, as well as the right and left eyes of the untreated group. One-way ANOVAs with post-hoc testing (Fisher’s least significant difference, LSD) were used to compare differences in gene expression levels between the eyes of lens-treated chicks and untreated chicks.

## Results

### Expression of TGFβs and Its Receptors in Normal Chick Ocular Tissues

Tissue samples from untreated chicks were used to establish baseline expression values for both the TGFβ isoforms and the TGFβ receptors in the three posterior ocular tissues. All three isoforms of TGFβ and also its three receptors were detected in all three ocular tissues—retina, RPE and choroid. While there are apparent tissue-specific differences in expression (Table 2), the nature of semi-quantitative PCR analyses precludes definitive conclusions.

**Table 2:** TGFβ isoform and TGFβ receptor gene expression profiles for retina, RPE and choroid from young normal (untreated) chicks, referenced to GAPDH.

Ocular		TGFβ isoforms			TGFB receptors	
Tissue	TGF-β1	TGF-β2	TGF-β3	TGFBR1	TGFBR2	TGFBR3
RPE	0.0169 ± 0.0030	0.0349 ± 0.0046	0.0169 ± 0.0022	0.0202 ± 0.0024	0.0015 ±0.0002	0.0102 ± 0.0029
Retina	0.0014 ± 0.0001	0.0063 ± 0.0006	0.0078 ± 0.0011	0.0027 ± 0.0001	0.00004 ±0.000007	0.0008 ± 0.00005
Choroid	0.0793 ± 0.0019	0.0439 ± 0.0029	0.1748 ± 0.0137	0.0264 ± 0.0036	0.0503 ±0.0075	0.0381 ± 0.0024

### Effects of Optical Defocus on Expression of TGF-βs and Receptors

#### Imposed myopic defocus

**Fig 1:**
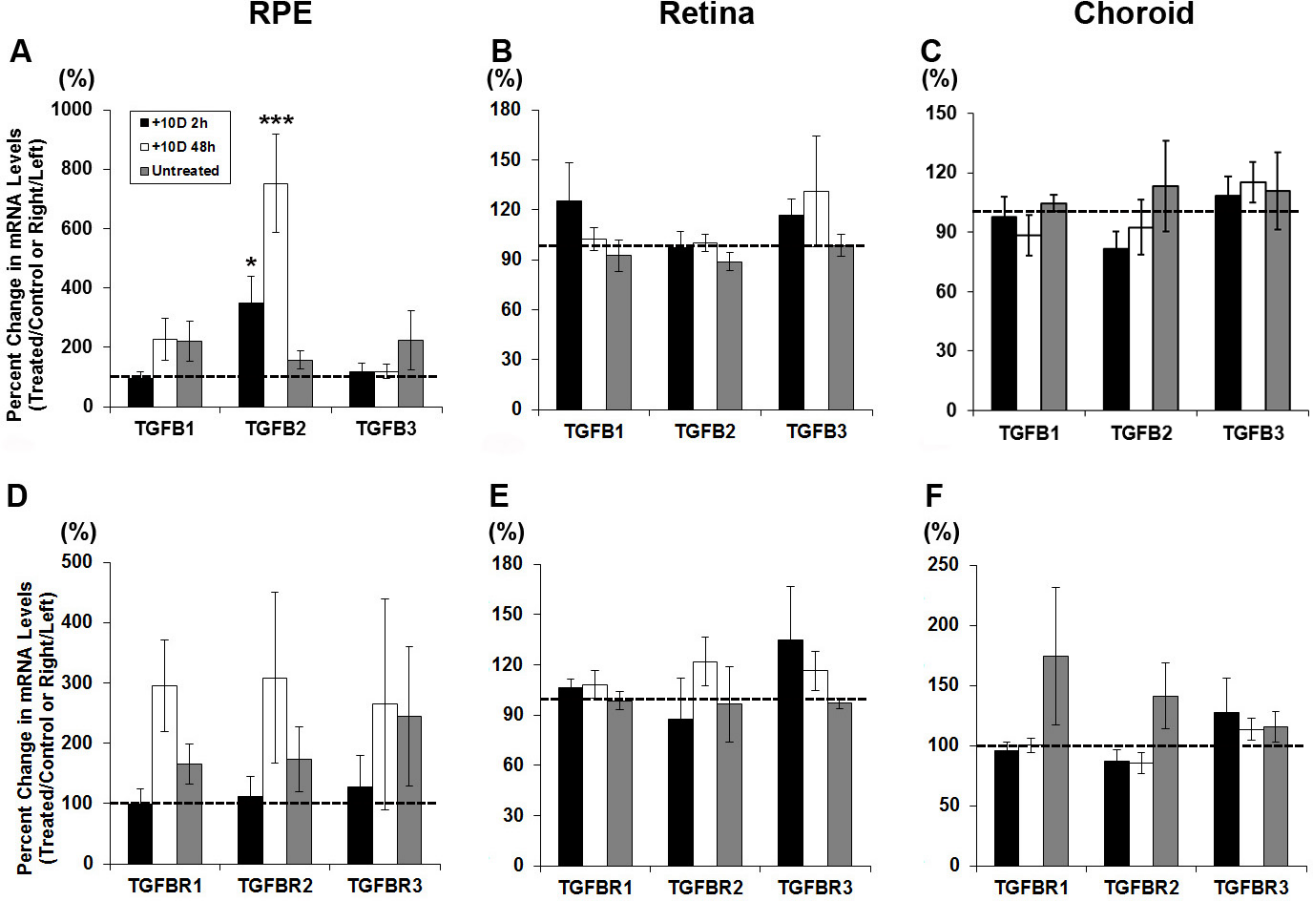
Percent change of gene expression for TGFβ isoforms (A, B, C) and TGFβ receptors (D, E, F), in RPE (A, D), retina (B, E), and choroid (C, F) from treated relative to fellow eyes of chicks wearing monocular +10 D lenses for 2 or 48 h; * *p* < 0.05; *** *p* < 0.001.

**Fig 2:**
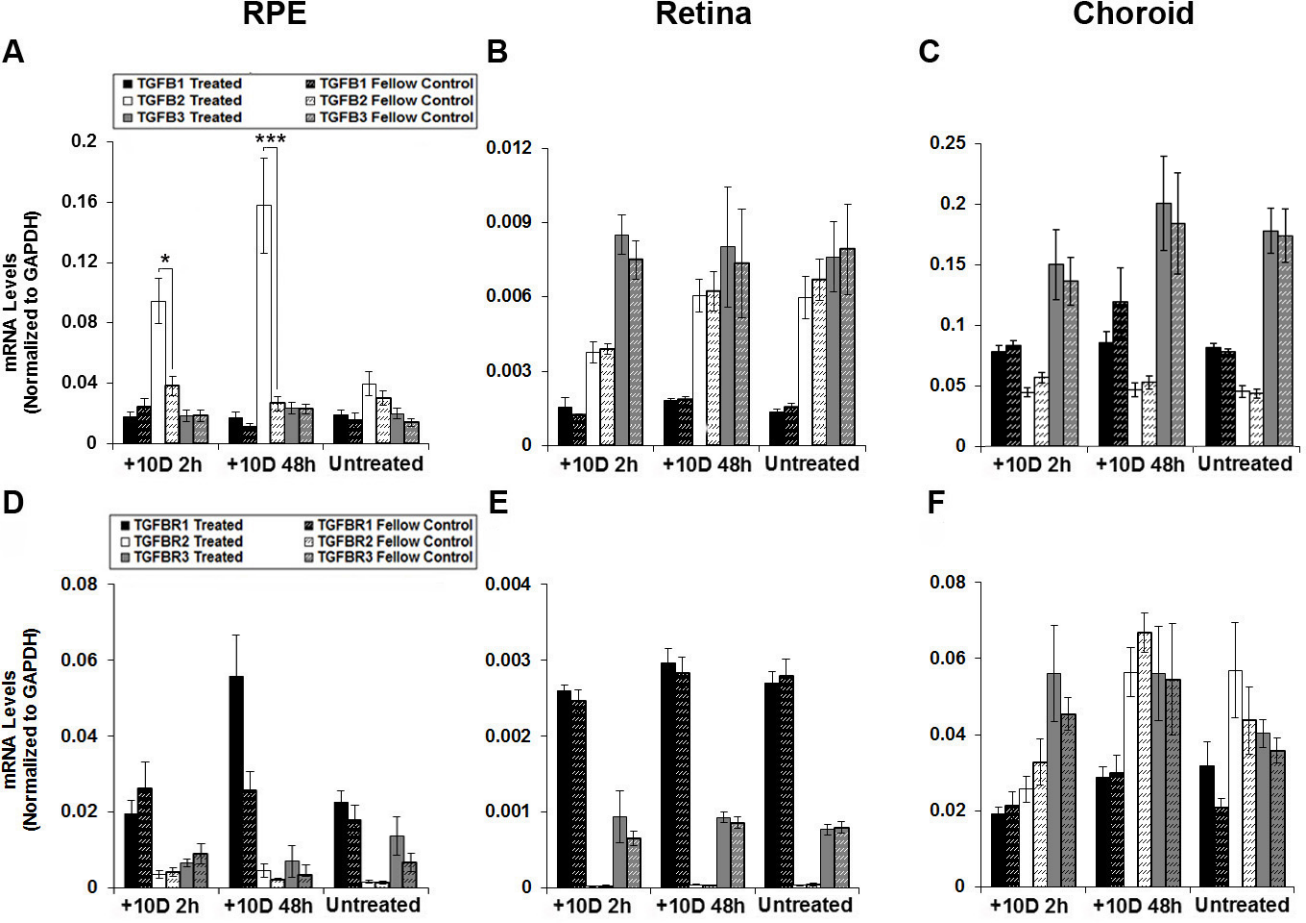
mRNA expression levels normalized to GAPDH for TGFβ isoforms (A, B, C) and TGFβ receptors (D, E, F), in RPE (A, D), retina (B, E), and choroid (C, F) from treated and fellow control eyes of chicks wearing monocular +10 D lenses for 2 or 48 h. Data from right and left eyes of untreated chicks shown for comparison. * *p* < 0.05; *** *p* < 0.001.

Exposure to myopic defocus (monocular +10 D lenses) resulted in selective up-regulation of just one gene, TGF-β2, in just one tissue, the RPE. Fig 1 shows the percent changes in mRNA expression in treated eyes relative to that in fellow (control) eyes for all six genes investigated and both 2 and 48 h time points, with expression levels normalized to GAPDH shown in Fig 2. In the RPE, TGF-β2 was significantly up-regulated after both 2 and 48 h exposures, more so with the longer exposure (349% increase ± 88%, *p* <0.05, n = 18; 752% ± 166%, *p* <0.001, n = 18; Fig 1A). As indicated above, none of the other genes showed significant, treatment-related, altered expression in RPE (Fig 1A–1D), although the result for TGFBR1 just failed to reach statistical significance (*p* = 0.06, n = 9). Furthermore, in the retina and the choroid, none of the genes showed significant, treatment-related, altered expression (Fig 1B, 1C, 1E and 1F). The same trends are evident in the normalized mRNA expression data (TGF-β isoforms, Fig 2A–2C; receptor isoforms, Fig 2D–2F).

#### Imposed hyperopic defocus

**Fig 3:**
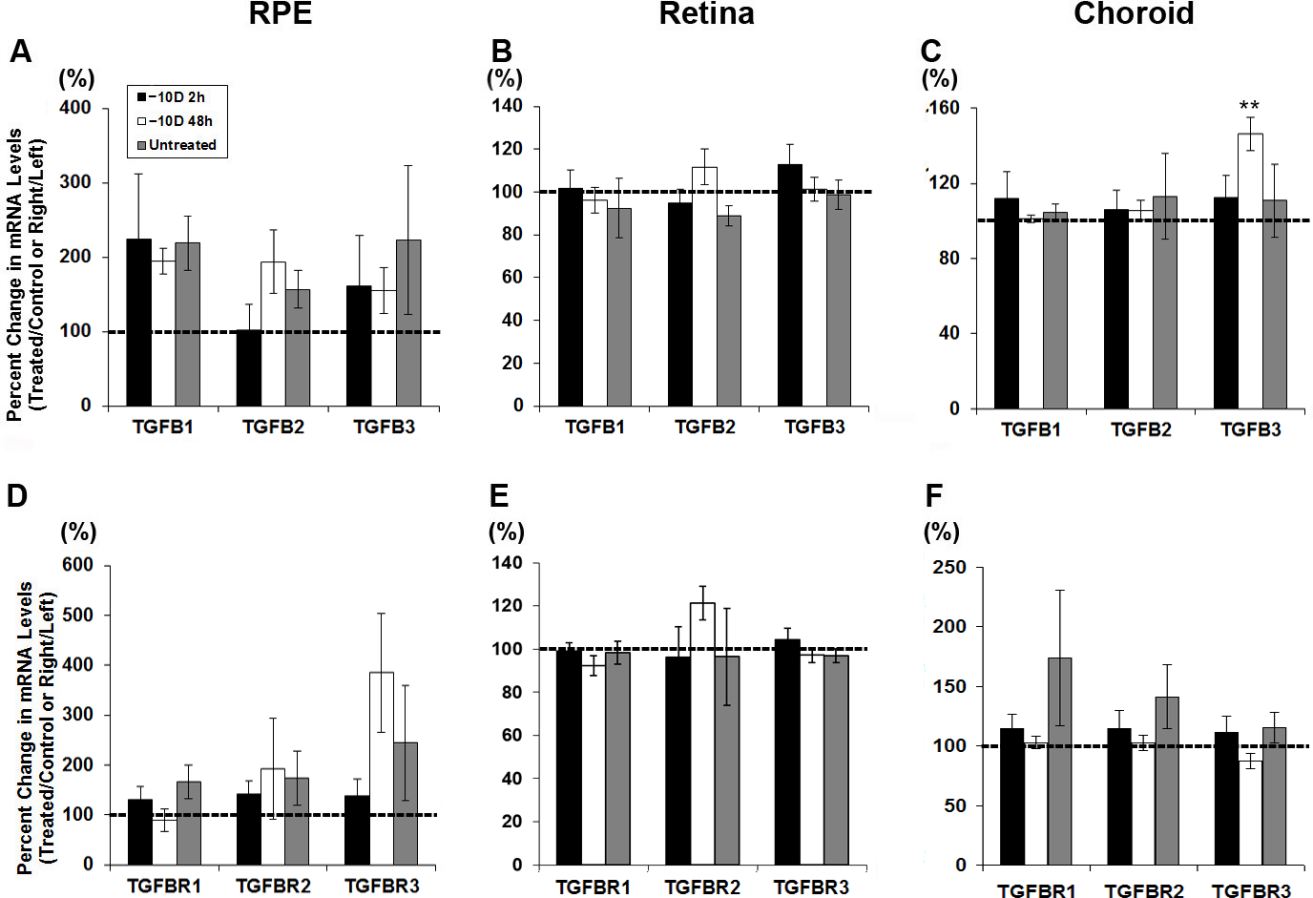
Percent change of gene expression for TGFβ isoforms (A, B, C) and TGFβ receptors (D, E, F), in RPE (A, D), retina (B, E), and choroid (C, F) from treated relative to fellow eyes of chicks wearing monocular -10 D lenses for 2 or 48 h; ** *p* < 0.01.

**Fig 4:**
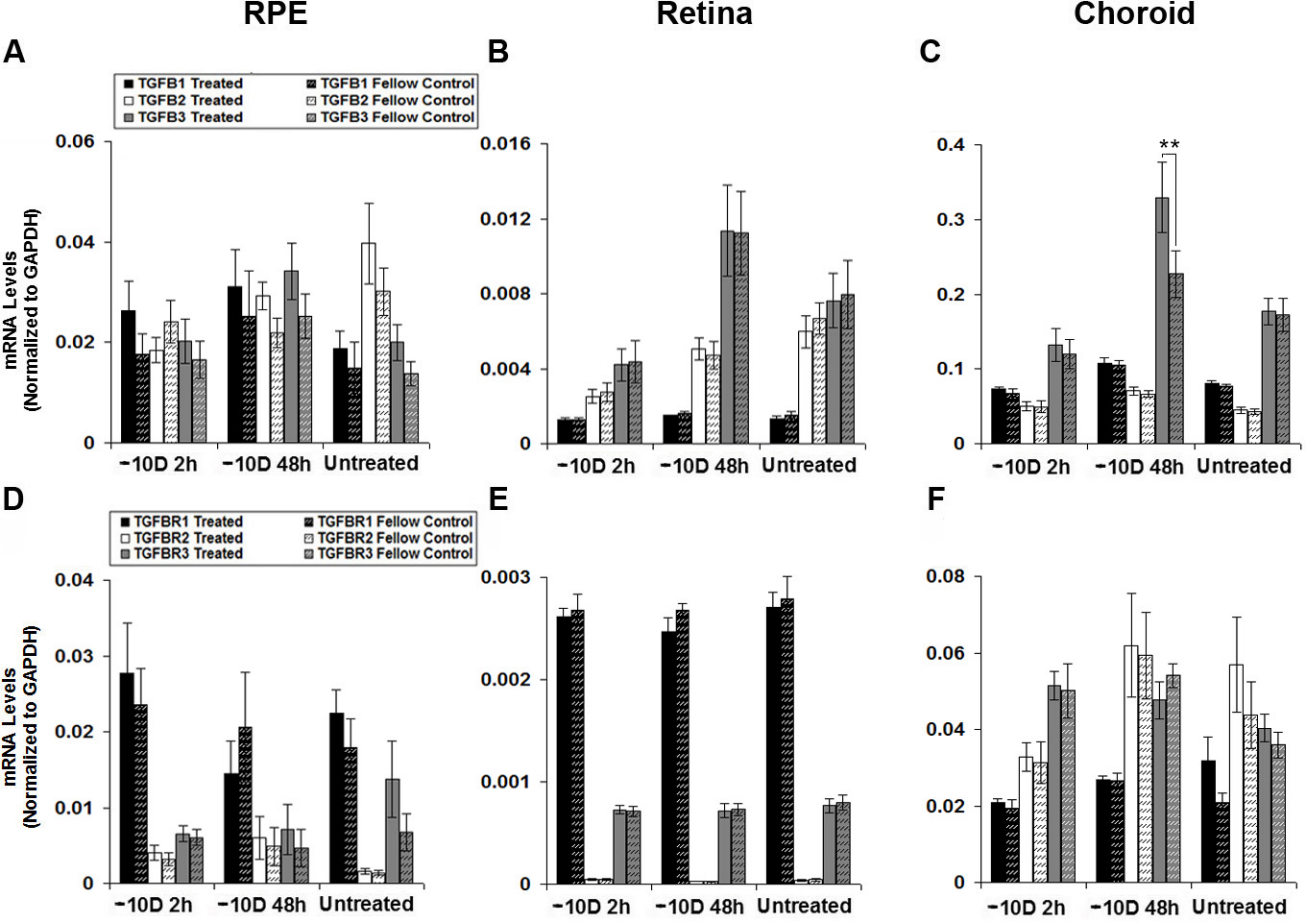
mRNA expression levels normalized to GAPDH for TGFβ isoforms (A, B, C) and TGFβ receptors (D, E, F), in RPE (A, D), retina (B, E), and choroid (C, F) from treated and fellow control eyes of chicks wearing monocular -10 D lenses for 2 or 48 h. Data from right and left eyes of untreated chicks shown for comparison. ** *p* < 0.01.

Exposure to hyperopic defocus (monocular -10 D lenses), also resulted in selective up-regulation of just one gene, in just one tissue, here TGF-β3 in the choroid. Fig 3 shows the percent changes in mRNA expression in treated eyes relative to that in fellow control eyes for all six genes investigated and both 2 and 48 h time points, and expression levels normalized to GAPDH are shown in Fig 4. In the RPE and retina, none of the six genes were significantly affected by the defocus treatment (Fig 3A, 3B, 3D and 3E), and in the choroid, the only exception was TGF-β3, which was significantly up-regulated relative to levels in fellow control eyes after 48 h of treatment (146% increase ± 9%, *p* < 0.01, n = 10; Fig 3C); neither of the other isoforms nor any of receptors showed altered expression in response to the defocus treatment (Fig 3F). Normalized mRNA expression data are consistent with these findings, the only result to reach statistical significance being that for choroidal TGF-β3, after a 48 h treatment period (Fig 4).

### Validation of GAPDH as a Reference Gene

**Fig 5:**
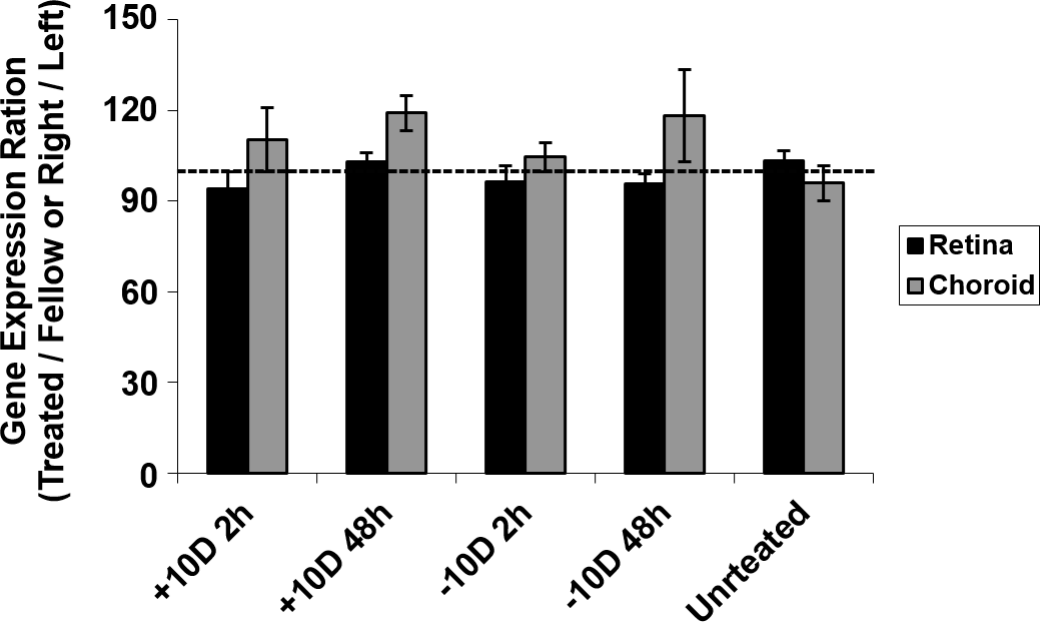
Stability of GAPDH expression across different treatment conditions in retina and choroid assessed by comparing the expression of GAPDH/total RNA (μg) from treated, fellow, and untreated eyes.

As in our previous study involving RPE,[24] GAPDH gene expression in retina and choroid proved to be stable across treatments. Neither differences in expression between treated and fellow eyes of treated chicks nor between the right and left eyes of untreated chicks reached statistical significance (Fig 5). Also, no yoking effect on gene expression of GAPDH was observed for any of the experimental manipulations.

## Discussion

In this study, we examined the gene expression in normal (untreated) chicks of the three TGFβ isoforms, TGF-β1, TGF-β2 and TGF-β3, and their three receptors, TGFBR1, TGFBR2, and TGFBR3, targeting the retina, RPE, and choroid, because of their assumed important roles in ocular growth regulation. The effects on expression of these genes of imposed optical defocus, the most widely-used experimental paradigm used to induce refractive errors, including myopia, were also investigated. Although TGFβs have been previously targeted in myopia research, studies have mostly targeted the sclera. The current study served to complement these previous studies. In normal, untreated eyes, we found all three TGF-β isoforms and all three TGF-β receptors to be expressed in all three key ocular tissues, although tissue-specific differences in expression were observed. We also documented tissue-specific, defocus-sensitive changes in gene expression, specifically involving TGF-β2 in the RPE, and TGF-β3 in the choroid. The significant up-regulation of TGF-β2 gene expression in RPE in response to imposed myopic defocus further supports our model of a retina-scleral cascade regulating ocular growth, in which the RPE plays a key role as a signal relay. The possibility that TGF-β2 serves as an inhibitor of ocular elongation, as suggested by these data, is also of interest in the context of myopia control.

How do our findings for normal chick eyes compare with results from the limited number of already published studies? Our finding that all TGFβ isoforms are expressed in chick retina is consistent with results of an earlier TGFβ protein expression study of retinas from 7 day-old chicks.[40] There are also both parallels and differences in the profiles reported here for the three isoforms of TGFβ and young chicks compared to observations from human, monkey, guinea pig, and tree shrew eyes.[30, 33, 36, 37, 39–46] Thus in tree shrew, TGF-β2 mRNA was reported to be most abundant in the retina, and levels of TGF-β1 and TGF-β2 mRNA were both higher than that of TGF-β3 in the choroid,[30] contrasting with the observations in chick, where TGF-β3 also proved to be highly expressed at the mRNA level in the retina as well as the most abundantly expressed isoform in the choroid. Expression levels for the RPE were not reported in this tree shrew study, nor were TGFβ receptors studied. In the current study, our gene expression data suggest the presence of all three TGFβ receptors in all three tissues—retina, RPE and choroid, at least in chick.

While a number of published studies, some involving the chick, have sought to understand the role of TGFβs in ocular growth regulation, their results have left many questions unresolved, in part because of the failure to isolate and separately study the components making up the wall of the eye and because of the varied approaches used to investigate this issue. Key results are summarized in Table 3, and reviewed here as background against which to view the results of our experiment involving short-term optical defocus. In the chick, the effects of form deprivation and negative lenses, both of which induce myopia, have been studied. In one of the earliest studies, form deprivation was reported to induce significant up-regulation of the TGF-β2 protein in both retina-RPE-choroid aggregates and sclera.[41] A follow-up study using immunohistochemistry to localize TGFβ reported intense staining in the posterior sclera of form-deprived eyes.[47] However, in one of two other closely related studies, form deprivation was reported to decrease the active but not latent forms of TGF-β proteins in retina-RPE-choroid aggregates and to decrease retinal photoreceptor layer immunoreactivity,[48] and in the second study, TGF-β2 protein immunoreactivity in retina and choroid was found to be unaffected by form deprivation and defocusing lenses.[40] Intense staining precluded analysis of treatment-related changes in TGF-β2 protein levels in the sclera in the latter study.[40] In terms of TGFβ gene expression, there are two chick studies of relevance, both involving imposed optical defocus to manipulate ocular growth. One study reported rapid and persistent down-regulation of TGF-β2 gene expression in the choroid after only 15 minutes of exposure to -7 D lenses, while no change of TGF-β2 gene expression was detected in retina.[49] The second study, which involved longer (4–72 h) exposures and both -7 and +7 D lens treatments, reported TGF-β2 mRNA to be increased after 4 h in the scleral cartilaginous layer of all lens-treated eyes, with this trend persisting out to 72 h in eyes treated with +7 D lenses.[33] Apart from the chick, the only other animal model to have been studied in this context is the tree shrew, which has a fibrous only sclera, as opposed to the bi-layered sclera of the chick. Reported changes in TGFβ expression in response to experimental manipulations in the tree shrew are also largely confined to the sclera. In form-deprived tree shrews, all three isoforms were reported to be down-regulated in the sclera over the first day of treatment, followed by a marked down-regulation of TGF-β2 compared to the other two isoforms between treatment days 1 and 5.[30] Similar trends were also reported in a more recent study, after myopia-inducing negative lens as well as form-deprivation treatments.[50] No changes in retinal and choroidal gene expression related to the development of myopia have been observed for any of the TGFβ isoforms in tree shrews,[39] although TGF-β2 was found to be down-regulated in the choroid of both treated and control eyes compared to values for untreated eyes in another study.[51]

**Table 3:** Summary of key findings from myopia-related animal model studies investigating the role of TGF-βs in eye growth regulation using either form deprivation (FD) or defocusing lenses to manipulate eye growth.

Animal	Visual Manipulation	Ocular Tissues	TGF-β Isoforms	Methods	Main Results	References
Chick	FD for 12 days	Retina-RPE-choroid	TGF-β2	Protein (ELISA)	↑	Seko Y, *et al*.,
		Sclera			↑	1995.[41]
Chick	FD for 10 days	Retina-RPE-choroid	TGF-β1	tRNA (PCR)	↓	Honda S, *et al*.,
		Retina-RPE-choroid	TGF-β1, 2, 3, 5	Active form of TGF-β protein (Western blot)	↓	1996.[48]
		Photoreceptor layer	TGF-β1, 2, 3, 5	Protein (immunohistochemistry)	↓	
Chick	+7 or -7 D lens for various periods of time	Retina/RPE	TGF-β2	mRNA (real-time PCR)	-	Simon P, *et al*.,
	(15, 30, 120 min)	Choroid			↓ with -7 D	2004.[49]
Chick	FD for 2 days;+7 or -7 D lens for various	Fundal layers-retina	TGF-β2	Protein (immunohistochemistry)	- (cell counting)	Mathis U, *et al*.,
	periods of time	Choroid			-	2010.[40]
	(40 min, 24 h)	Sclera			No quantitative analysis	
Chick	FD for 14 days	Sclera	TGF-β1, 2, 3, 5	Protein (immunohistochemistry)	↑	Kusakari T, *et al*., 2001.[47]
Chick	+7 or -7 D lens for various periods of time	Cartilaginous sclera	TGF-β2	mRNA (real-time PCR)	↑ with +7 D	Schippert R, *et al*.,
	(4, 24, 72 h)	Fibrous sclera			-	2006.[33]
Tree	FD for various periods	Retina	TGF-β1, 2, 3	mRNA (real-time PCR)	-	Jobling A,
shrew	of time (3, 7, 11, 24 h, 5 days)	Retina	TGF-β2	Protein active/latent ratio (ELISA)	↓	*et al*., 2009.[39]
		Choroid	TGF-β1, 2, 3	mRNA (real-time PCR)	-	
Tree shrew	-5 D lens or FD for 2 days	Choroid	TGF-β2, 3	mRNA (real-time PCR)	-	He L, *et al*., 2014.[51]
Tree shrew	FD for 1 or 5 days	Sclera	TGF-β1, 2, 3	mRNA (real-time PCR)	↓	Jobling A, *et al*., 2004.[30]
Tree shrew	-5 D lens or FD for various periods of time (2, 4 days)	Sclera	TGF-β1, 2	mRNA (real-time PCR)	↓	Guo L, *et al*., 2013.[50]

Form-deprivation, FD

Lens-induced myopia, LIM

Lens-induced hyperopia, LIH

↑, increased treated compare to control

↓, decreased treated compare to control

-, no change treated compare to control

Our study was designed to separately analyze changes in expression in retina, RPE and choroid of TGF-β isoforms and its receptors. Our choice of short optical defocus exposure times, i.e., of 2 and 48 h, aimed in the first case to allow adequate time for the imposed defocus to be processed by the retina, and in the second case, to allow the generated growth modulatory signal to be relayed to the choroid, as evidenced by changes in its thickness. By avoiding longer exposure times, we sought to avoid gene expression changes that may occur secondary to dimensional changes, for example, due to altered biomechanical forces on the walls of the eye. Significant treatment-induced gene expression changes in our study were limited to: 1) +10 D lens-induced significant up-regulation of TGF-β2 in the RPE, and 2) -10 D lens-induced up-regulation of TGF-β3 in choroid. Both findings add weight to evidence implicating TGFβs in eye growth regulation and further implicate the RPE and choroid in the retina-to-sclera signaling cascade, although the specific mechanism(s) by which TGFβs effect changes in choroidal thickness and in the rate of ocular elongation and thus eye size remain to be fully understood.

The findings in our study of defocus-induced altered expression of TGF-β2 in the RPE and TGF-β3 in choroid raise the question of whether the levels of related protein are also altered and if so, what are their likely effects. Relevant research has mostly focused on scleral cells as potential targets, using cell and tissue culture paradigms. Key studies and results are summarized in Table 4. In culture, TGFβs have been shown to cause morphologic changes in both chick scleral chondrocytes and fibroblasts.[52] In studies involving cultured tree shrew scleral fibroblasts, suitably designed cocktails of TGFβs to mirror form deprivation-induced scleral gene expression changes for the three isoforms were found to reduce the synthesis and secretion of proteoglycans and production of collagen, simulating the scleral changes documented *in vivo* in myopic eyes.[30, 31, 53] The latter represent key features of scleral extracellular matrix remodeling, as underlies altered eye elongation in eyes with fibrous only scleras.[53] Similar cocktails of TGFβ isoforms also promoted cell contraction and induced the rapid differentiation of cultured scleral fibroblasts into α-SMA-expressing myofibroblasts.[18, 31, 53]

**Table 4:** Summary of key findings of studies investigating the effects of TGF-βs on scleral cell and tissue cultures.

TGF-β Treatment	Cell or Tissue Culture	Treatment Effect Tested	Methods	Main Results	References
TGF-β	Primary scleral chondrocytes & fibroblasts	Cell proliferation	Cell counting & morphological changes	↑	Seko Y, *et al*., 1995.[52]
TGF-β (mixture)	Chick tissue culture	Cellular proliferative activity	[^3^H] thymidine incorporation	↓	Honda S, *et al*., 1996.[48]
TGF-β1, 2, 3	Primary tree shrew scleral fibroblasts	Collagen production	[^3^H] proline incorporation	↑	Jobling A, *et al*., 2004.[30]
TGF-β1, 2, 3	Primary tree shrew scleral fibroblasts	Cell-mediated contraction & scleral cell phenotype alteration (α-SMA-expressing myofibroblasts)	mRNA (real-time PCR) Protein (immunocytochemistry)	↑	Jobling A, *et al*., 2009.[53]

↑, increased treated compare to control

↓, decreased treated compare to control.

Of *in vivo* animal myopia studies involving TGFβ treatments, one of relevance to the current study reported that subconjunctival injection of TGF-β1 inhibited the myopia rescue effect of bFGF in the chick form-deprivation model.[54] The direction of the latter effect of TGF-β1 tends to rule it out as a candidate for myopia control. Nonetheless, our finding of defocus-induced increased gene expression of TGF-β2 in chick RPE, linked to slowed eye elongation, and the above *in vitro* tree shrew scleral data raise the possibility that this isoform of TGFβ might have application as a myopia control therapy. Studies are underway to address the question of whether such mRNA expression changes are translated into increased protein expression. Nonetheless, it is plausible that TGF-β2 protein secreted from the basal side of the RPE could act on the adjacent choroid to trigger another step in the signal transduction cascade and/or diffuse to the sclera to regulate its remodeling after activation.[48, 55] Either way, the RPE and TGF-β2 would seem plausible targets for controlling myopia. That TGF-β3 gene expression showed an increase with myopia-inducing negative lenses in the nearby choroid opens the possibility of more than one signal pathway being involved, perhaps targeting different tissues.

In earlier studies, we reported defocus-induced changes in RPE gene expression in the chick for three other members of the TGF-β superfamily, BMP2, BMP4, and BMP7. All showed differential gene expression in response to short-term lens treatments (+10 and -10 D lenses).[24, 25] It remains unclear how these growth factors are regulated in RPE, although presumably one or more retinal signals are involved. It is also unclear how these various growth factors interact. The possibility of cross-talk downstream, between activated signaling pathways, with the further possibility of synergistic effects on eye growth regulation, cannot be ruled out. For example, co-expression of TGFβ and BMP receptors has been described in developing chick retina and Smad-dependent pathways downstream to TGF-β and BMP share a common component, Smad4.[56, 57] TGFβ and BMP isoforms also activate some of the same transcription factors, including TGFβ-inducible early gene (TIEG), and share the same transcriptional co-activator, GCN5.[58–60] Finally, both TGFβ and BMP signaling pathways interact with Notch, Wnt, and STAT pathways.[57, 61]

In the current study, no defocus-related changes in gene expression were detected in retina for any of the TGFβ isoforms (TGF-β1, 2, 3), and likewise, the expression of TGF-β1 and 2 in choroid were apparently unaffected by defocus. These results closely parallel findings in tree shrew.[39] Nonetheless, since both retina and choroid comprise many different cell types, we cannot rule out the possibility that a sparse subpopulation of cells within one or both tissues did undergo altered (differential) expression of one or more of the genes investigated. Only investigations at the cellular level, for example, using laser capture microdissection,[62] can eliminate this possibility.

In summary, we report here broad distribution at the gene expression level of all three TGFβ isoforms and their receptors, specifically in the three layers making up the posterior eye wall of young chicks, and in addition, differential gene expression of TGF-β2 in RPE and TGF-β3 in choroid, in response to imposed defocus. These findings represent further supporting evidence for roles in ocular growth regulation of members of the TGFβ family and the involvement of RPE as a critical relay station in the retina-scleral signaling cascade mediating eye growth regulation. Further investigations into related molecular mechanisms may allow the application of one or more of these growth factors as novel treatments for controlling myopia.

## Supporting Information

S1 Table Gene expression levels for all gene tested. (XLSX)
